# Experimental West Nile virus infection provides lessons for recovery from enteric neuropathies

**DOI:** 10.1172/JCI185865

**Published:** 2024-11-01

**Authors:** Joel C. Bornstein

**Affiliations:** Department of Anatomy and Physiology, University of Melbourne, Parkville, Victoria, Australia.

## Abstract

Loss of enteric neurons leading to long-term gastrointestinal dysfunction is common to many diseases, and the path to functional recovery is unclear. In this issue of the *JCI*, Janova et al. report that West Nile virus killed enteric neurons and glia via CD4^+^ and CD8^+^ T cells acting through the perforin and Fas ligand pathways. Enteric glial cells contributed to neurogenesis and at least partial replacement of affected neurons. While neurogenesis is important for recovery, dysmotility and disruptions to the network structure persisted. Following enteric injury, the contribution of neurogenesis and the conditions that support restoration of enteric neural circuits for functional recovery remain for further investigation.

## T cells mediate neuron death and dysmotility

Many infectious diseases alter intestinal functions. Symptoms of such diseases often include diarrhea and constipation that are associated with changes in the enteric nervous system (ENS), which regulates the propulsion of content within the gastrointestinal tract. In this issue of the *JCI*, Janova et al. (1) explored mechanisms by which West Nile virus (WNV) produced dysmotility in mice as a model for infectious insults to the gut. The authors built on their earlier work implicating T cells in the death of enteric neurons leading to dysmotility (2). Janova and authors showed that adult neurogenesis followed the acute response to infection (1). These findings suggest that neurogenesis may be the key to recovery from all neurotoxic change to the ENS, whether triggered by infection, cancer chemotherapy (e.g. oxaliplatin) (3), inflammation (4), or other insults (5). Typically, enteric neurotoxicity leads to persistent dysfunction, so initiation of neurogenic recovery is vitally important for human health and quality of life.

Janova et al. (1) used intersecting approaches involving transgenic mice and blocking antibodies. WNV infected enteric neurons and glia, leading to cell death induced by helper (CD4^+^) and cytotoxic (CD8^+^) T cells. By post-infection day seven, whole intestinal transit time, neuron numbers, and the density of neural and glial networks were affected. The authors systematically explored the roles of both T cell classes, showing that other immune cells (neutrophils, B cells, nonresident macrophages/monocytes) had little or no role in the response to WNV (1). This finding contrasts with other viruses, such as herpes simplex virus, which induces various immune cells (6, 7). Notably, resident macrophages reduced damage (1) and have been proposed in other contexts to provide neuroprotection (8). Given that nonresident macrophages accumulated within affected ganglia, the mediators of pathogen-induced neurotoxicity probably did not reflect the changes in immune cell numbers. Notably, the mechanism by which the T cells produced injury predominantly involved cytotoxic and death receptor pathways, via perforin and Fas ligand, respectively (1).

The loss of neuron cell bodies due to WNV appears to be relatively nonspecific, as both myenteric and submucosal plexus neurons are affected, with major neuronal subtypes, including calretinin- and nNOS-positive neurons, being equally vulnerable. Loss of diverse neuronal groups probably accounts for dysmotility, as cell bodies are where input from other neurons is integrated, so their death blocks physiological action potential signaling, even though the decentralized axons persist for several days. Thus, dead neurons would be unable to participate in network activity, or contribute output to smooth muscles, or activate other effector tissues. Since some calretinin neurons are excitatory motor neurons and most nNOS neurons are inhibitory motor neurons (5), neural control of the smooth muscle becomes compromised and is likely to produce localized strictures with proximal accumulation of content where nNOS innervation is particularly affected (1). Such strictures substantially delay transit.

It should be noted, however, that loss of submucosal neurons (1) is expected to result in dysfunction due to disrupted neurogenic water and electrolyte secretion, rather than dysmotility. Furthermore, general loss of enteric neurons would alter neurally driven immune activity, mucus secretion, and epithelial cell production in the crypts, among many other functions (5).

Loss of cell bodies has other, less straightforward effects by depriving surviving neurons of some synaptic targets and depriving others of inputs. This synaptic loss may have retrograde effects on surviving neurons and lead to rewiring within the remaining circuit. However, there are many different functional subtypes of neurons mixed together within any myenteric ganglion (5), so axons of surviving neurons will have a variety of synaptic targets in different intermingled enteric circuits from which to choose. Thus, new connections may aid or impede the recovery of function depending the appropriateness of these synapses. Whether such opportunistic synaptogenesis occurs within a damaged ENS is unknown, but is a key question for all enteric neuropathies (9).

## Neurogenesis alone does not restore function

In the study by Janova et al., the quantity of neurons, and specifically calretinin and nNOS neurons, recovered with time after infection (1), indicating marked neurogenesis. Neurogenesis in the adult ENS has been shown to be triggered by chemical injury (10) or inflammation (11), with the new neurons being derived from enteric glial cells that serve as a reservoir of precursors. These reservoir cells may be a subset of glia that express VMAT2 (encoded by *Slc18a2*) (12), although this remains to be demonstrated conclusively. Janova and co-authors showed that, despite the addition of newborn neurons after WNV infection, network densities, which reflect the balance of surviving, degenerating, and growing axons and dendrites (i.e., the wires in the circuit), and intestinal transit did not recover over the same time period (1). Thus, the formation of new synapses by surviving neurons was insufficient to restore network function, showing that complete neural circuits are needed to produce proper function.

**Figure 1:**
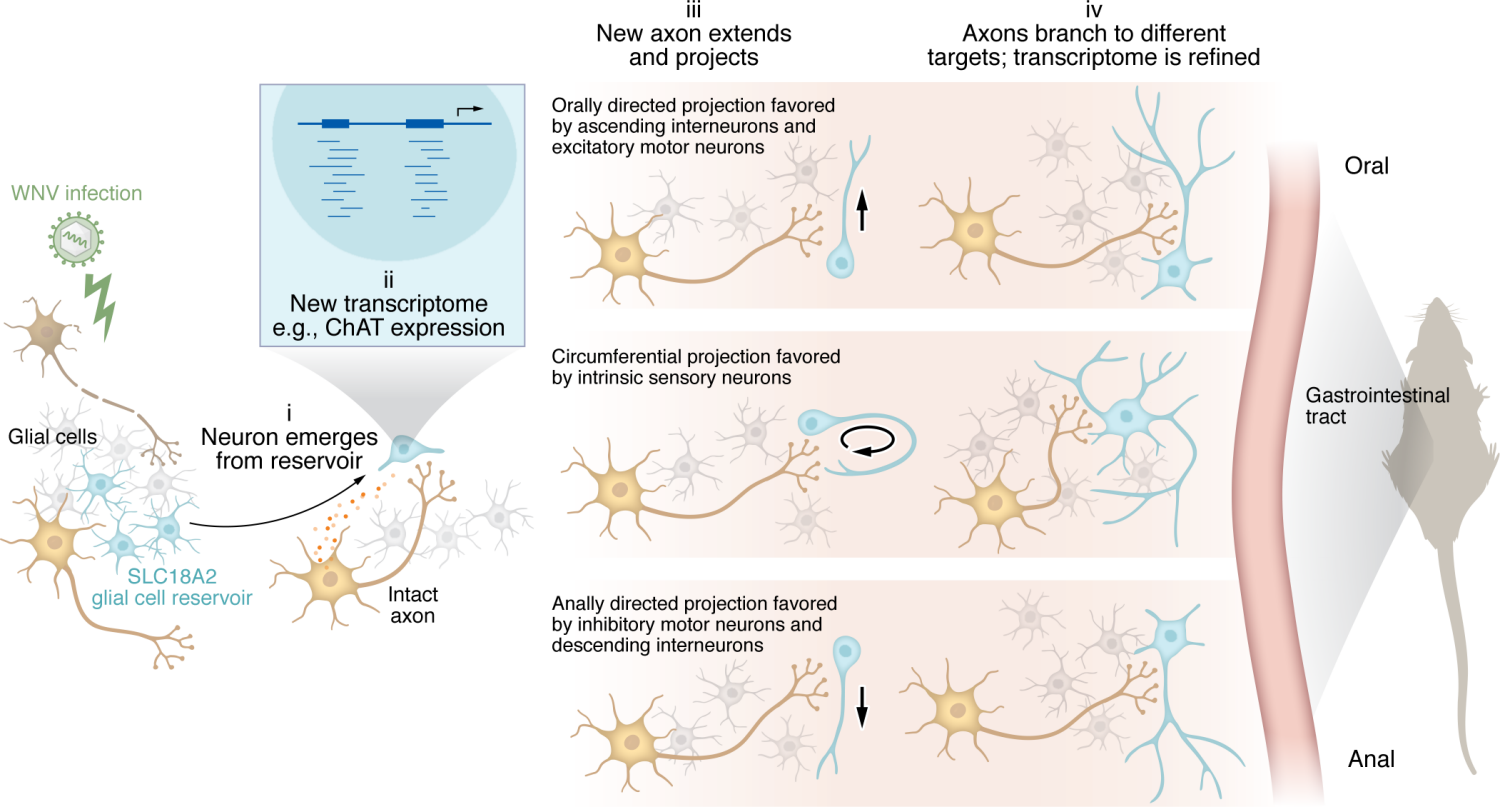
After injury, several events and processes may contribute to the phenotypic differentiation of newborn enteric neurons in an adult ENS. (i) Following injury, such as WNV infection, new neurons arise from reservoir cells, likely SLC18A2 glial cells, and receive contacts from intact axons in specific reflex pathways, usually within days. (ii) Input, whether synaptic or trophic, from these new synapses regulates the generation of a transcriptome consistent with the new neuron becoming part of the functional circuit served by the intact axon. ChAT, choline acetyltransferase. (iii) The new transcriptome sets the preferred projection direction of the new axon, with orally directed outgrowth being favored by ascending interneurons and excitatory motor neurons, intrinsic sensory neurons projecting circumferentially, and inhibitory motor neurons and descending interneurons extending anally. (iv) The glial scaffold facilitates branching of enteric axons as they diverge to their different targets (18), interneurons supply neurons in other ganglia over projection distances of more than 1 cm, and motor neurons enter the muscle, where they branch extensively. Synaptic connections are made between the original survivor and newborn neurons in the relevant pathway, selected by their expressed transcriptome. Activity in the pathway and retrograde feedback from downstream targets allow refinement of the transcriptome to its mature form.

A key question for functional recovery via neurogenesis after death of enteric neurons involves determining the necessary components for the neurochemical differentiation of newborn neurons. In this context, differentiation of neurons occurs in a relatively intact system where they are surrounded by multiple neuronal subtypes and several types of glia. Single-cell RNA-Seq (scRNA-Seq) has identified 12 to 14 different neuronal subtypes in the myenteric plexus of the mouse small intestine (13, 14). Phenotypic differentiation during ENS development in utero and postnatally depends on systematic progression, with some neurochemical phenotypes emerging early and influencing the differentiation of other subtypes (15). This progression has clear branch points, and Morarach et al. (13) identified distinct branches of diversification of enteric neural crest derivatives between E15.5 and E18.5 using scRNA-Seq. How this process transpires in a mature, but depleted, ENS is a major consideration for recovery after diffuse lesions or developmental delays (16), as cues that inform fate decisions may be absent or masked by other signals. Does the neurochemical phenotype of a neuron depend on feedback from its targets or its synaptic inputs? It is possible that new synapses from surviving axons in specific neural pathways program a new neuron to express an appropriate transcriptome to ensure discrimination between interneurons and motor neurons (Figure 1).

## Challenges to reestablishing circuits and therefore function

For functional recovery, newly differentiated neurons must send out axons to find appropriate targets. As functionally distinct neurons project in different directions, a key step involves the directionality in which the projection will align. Intrinsic sensory neurons wrap around the gut wall circumferentially, while excitatory motor neurons and some interneurons align orally toward the mouth, and inhibitory motor neurons plus other interneurons direct their projections toward the anus (5). Related questions also warrant consideration: What features identify new neurons as suitable targets, and which original survivors or immature neurons might serve as suitable partners given the plethora of functions mediated by enteric neurons? There are some data addressing these questions from the transplantation of progenitor-derived neurospheres into an intact ENS. These neurospheres generate functional inhibitory and excitatory motor neurons plus some cholinergic interneurons that are innervated by the native neural circuits (17). Excitatory motor neurons differentiate and innervate circular smooth muscle within two weeks of transplantation (18), indicating that reestablishing circuit connections may be the rate-limiting process for the diffuse lesions, similar to those produced by WNV. Intrinsic sensory neurons are notably absent from these newly differentiated neurons, and whether all subtypes of interneurons develop also remains unknown. In mice, the synaptic targets can be over 1 cm away, (19) meaning that the axons pass through more than 40 myenteric ganglia, making some contacts along their route. So there will be multiple alternative synaptic partners along the way, including other newly differentiated neurons and surviving adult neurons in different functional pathways that have lost some of their inputs. The siren call of inappropriate partners must be ignored, but how?

Some issues for circuit rewiring have very recently been addressed. Stavely et al. (18) elegantly showed that newly differentiated neurons integrate into intact ENS by sending axons along surviving glial pathways. Furthermore, glia were found to promote branching of neurites from newly differentiated neurons, thereby facilitating the extensive divergence of neural outputs essential for integration in the enteric circuits (18). These findings suggest a further explanation for the delayed recovery of network density after WNV, beyond a simple reduction in the quantity of glia (1). Fewer glia may also reduce the precursor pool for neurogenesis, impair scaffolding that supports axon arborization (18), and retard restoration of the neural networks in the ganglionated plexuses and the muscle.

There is much we don’t know about recovery of function after neurotoxic insults to the ENS. Neurogenesis is essential, but the mechanism by which axon extension and synaptogenesis restore connectivity in the circuit and how we can facilitate them are issues crying out for investigation.

## Version 1 11/01/2024

Electronic publication
